# Species delimitation and mitonuclear discordance within a species complex of biting midges

**DOI:** 10.1038/s41598-022-05856-x

**Published:** 2022-02-02

**Authors:** Phillip Shults, Matthew Hopken, Pierre-Andre Eyer, Alexander Blumenfeld, Mariana Mateos, Lee W. Cohnstaedt, Edward L. Vargo

**Affiliations:** 1 Department of Entomology, Texas A&M University, College Station, TX 77843 USA; 2 USDA, APHIS, Wildlife Services, National Wildlife Research Center, Fort Collins, CO 80521 USA; 3 Department of Ecology and Conservation Biology, Texas A&M University, College Station, TX 77843 USA; 4 USDA-ARS Arthropod Borne Animal Disease Research Unit, 1515 College Ave, Manhattan, KS 66502 USA

**Keywords:** Molecular ecology, Population dynamics, Entomology

## Abstract

The inability to distinguish between species can be a serious problem in groups responsible for pathogen transmission. *Culicoides* biting midges transmit many pathogenic agents infecting wildlife and livestock. In North America, the *C. variipennis* species complex contains three currently recognized species, only one of which is a known vector, but limited species-specific characters have hindered vector surveillance. Here, genomic data were used to investigate population structure and genetic differentiation within this species complex. Single nucleotide polymorphism data were generated for 206 individuals originating from 17 locations throughout the United States and Canada. Clustering analyses suggest the occurrence of two additional cryptic species within this complex. All five species were significantly differentiated in both sympatry and allopatry. Evidence of hybridization was detected in three different species pairings indicating incomplete reproductive isolation. Additionally, COI sequences were used to identify the hybrid parentage of these individuals, which illuminated discordance between the divergence of the mitochondrial and nuclear datasets.

## Introduction

Speciation is a dynamic evolutionary process through which populations segregate into independently evolving lineages over time^1^. When gene flow is restricted either through geographic, behavioral, or ecological isolation, the accumulation of genetic changes, through selection or local genetic drift, may lead to divergence and potentially reproductive isolation^2–6^. Thus, the amount of genetic differentiation and level of gene flow between closely related lineages can be used to evaluate the strength of this isolation and determine species status^7^. Depending on the completeness of the speciation process between lineages, it can be challenging to unambiguously identify species^8^. Shallow divergence and hybridization can mask both morphological and genetic differences. While the most accurate assumptions about species delimitation are derived from a multifaceted approach^9,10^, genomic data has become a powerful tool to investigate species boundaries^11,12^. Both substantial and fine-scale genetic divergence is being uncovered across many study systems, even in the absence of morphological variation^13–16^. Species delimitation is especially important when working with organisms responsible for pathogen transmission, as misidentifications will lead to inaccurate vector surveillance data. *Culicoides* Latreille (Diptera: Ceratopogonidae) biting midges are responsible for the transmission of many pathogens worldwide^17,18^, including bluetongue virus (BTV) and epizootic hemorrhagic disease virus (EHDV). These viruses can cause severe symptoms and death in wild and domestic ungulates and are responsible for substantial economic losses globally^19,20^.

In North America, one of the main BTV and EHDV vectors is *Culicoides sonorensis* Wirth and Jones^20^, which belongs to the *C. variipennis* species complex. When originally described, this group consisted of five subspecies^21^; though presently, three distinct species are recognized (*C. occidentalis* Wirth and Jones, *C. sonorensis*, and *C. variipennis* (Coquillet)) with *C. albertensis* Wirth and Jones and *C. australis* Wirth and Jones designated as synonyms of *C. sonorensis*^22^. Despite the current taxonomic arrangement, species identification remains difficult due to very subtle morphological differences and overall genetic similarities^23,24^. Additionally, the presence of cryptic species or hybridization within the complex could be further complicating proper species identification. Under laboratory conditions, *C. sonorensis* and *C. occidentalis* have been shown to hybridize^25^, and both *C. occidentalis* and *C. variipennis* occur sympatrically with *C. sonorensis*^21^. Hybridization with *C. sonorensis* in nature would represent a pathway for introgression, potentially for genes controlling vector competency^26^.

Geographic isolation limits gene flow between populations, and thus, life-history traits influencing dispersal ability can drastically influence the level of gene flow among populations (e.g.^27,28^). Species with low dispersal ability are particularly likely to exhibit highly differentiated populations resulting in the evolution of cryptic species over a limited spatial scale^29^. In contrast, species with high dispersal abilities are likely to maintain a high level of gene flow between populations. Studies of *Culicoides* species in Europe, Africa, and Australia have consistently revealed frequent gene flow between populations, even at continental scales^30–33^. *Culicoides* species have been shown to randomly disperse away from their larval habitats, up to 2 km daily^34,35^, and are also known to disperse via prevailing winds for hundreds of kilometers^36–38^. The high dispersal ability of biting midges decreases the likelihood of geographic isolation between populations, and as a consequence, may not have played a major role in the diversification of this group. Instead, ecological or behavioral isolation can allow closely related species to occur sympatrically in distinct ecological niches, and may help explain species divergence within *Culicoides*^39,40^. The high rate of dispersal, potential for hybridization, and numerous sympatric populations make the *C. variipennis* complex an intriguing system in which to study species delimitation and may also provide insights into the mechanisms responsible for speciation within this group.

Here, we evaluated the genetic structure of the *C. variipennis* complex from broad-ranging sampling locations to test the current taxonomic hypothesis of three distinct species. We used ddRadSeq to analyze 206 individuals collected from 17 sites throughout the United States and Canada. We first estimated the overall genetic similarity and population structure among these samples to delimit distinct lineages within the species complex. We then estimated the level of gene flow within and between the inferred species. As previous attempts to separate these species using common barcoding genes have been inconclusive, we sequenced a region of the COI gene to compare to the putative SNP species identifications. Additionally, we discuss the potential mechanisms controlling reproductive isolation within this species complex.

## Results

### SNP calling and clustering analyses

**Figure 1:**
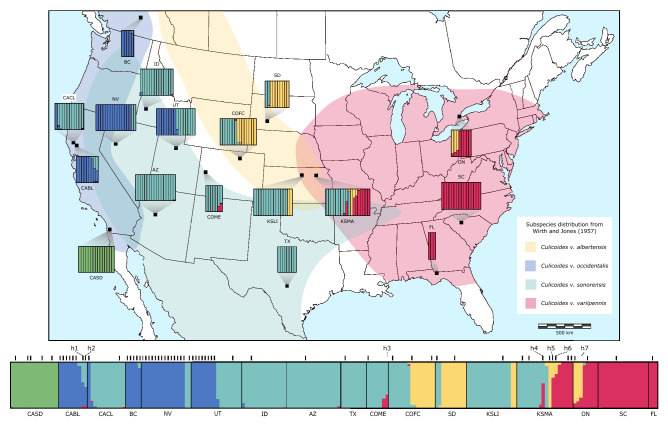
Geographic distribution and structure plots for each collection site (black squares) overlaid on the historical distribution of the species described in Wirth and Jones 1957. The fastSTRUCTURE results are for 206 individuals inferred by 3612 SNPs and assuming five populations (K = 5). The vertical bars within each collection site represents an individual, with each color representing a cluster. The putative species identity of each cluster are as follows: *Culicoides occidentalis* (blue), *C. sonorensis* (teal), *C. albertensis* (yellow), *C. variipennis* (red), and an unidentified population in San Diego, CA (CASD) (green). The black bars above the overall structure plot indicates an individual for which the COI gene was also sequenced. The individuals inferred to be hybrids are labeled h1-7. This map was created using Inkscap v.1.1 (https://inkscape.org/).

**Figure 2:**
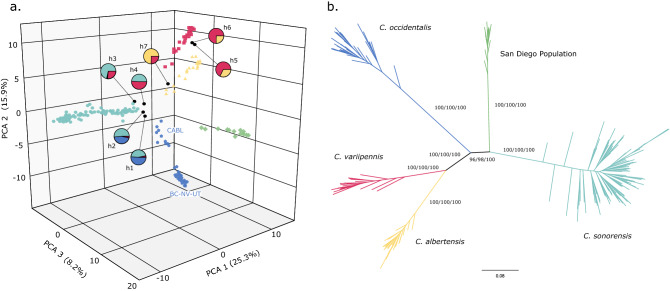
(**a**) A 3D representation of the principal component analysis (PCA) of all individuals included in the study. Each color represents the cluster inferred from the structure analysis; *C. albertensis* (yellow), *C. occidentalis* (blue), *C. sonorensis* (teal), *C. variipennis* (red), and the unidentified San Diego population (green). Hybrids (h1–h7) are designated with a black circle and their inferred parental ancestry is depicted with pie charts. The geographic locations of the two *C. occidentalis* clusters are labeled next to each grouping (see Table 1 for abbreviation). (**b**) Unrooted maximum likelihood phylogenetic tree based on 199 individuals inferred from 3612 SNPs (the hybrids were removed here but are included in Fig. S3.). Clade colors also represent the clusters inferred from the structure analysis. Support values written on the branches: rapid bootstrap (%)/SH-aLRT support (%)/ultrafast bootstrap support (%). For clarity, the values within each cluster are not shown.

In total, 271 individuals were subjected to the ddRADseq procedure and yielded an average of 2.08 million reads per individual. During the initial filtering, 36 individuals were found to have low-quality sequences (phred score of less than 25) and were removed from the dataset. Additionally, 29 individuals were found to have more than 75% missing data and were also removed. The final dataset included 206 individuals from 17 sites and contained 3612 SNPs. The population structure inferred by fastSTRUCTURE that best explains the data was K = 5. Structure plots showing K = 3–6 can be found in Figure S1. At K = 5, most individuals (86%) were unambiguously assigned to one group (98–100% assignment score; Fig. 1). Consistent with these results, the principal component analysis (PCA) and discriminant analysis of principal components (DAPC) grouped these individuals into five main clusters (Figs. 2a & S2). The main difference being that the PCA further segregated one cluster (blue, Fig. 2a) into two separate groups; east and west of the Sierra Nevada mountain range. Further support for the same five clusters was found in the maximum likelihood trees, with a high level of support from each approximation method (Figs. 2b & S3).

### Inter- and intra-species population genetics

The geographic distributions of these clusters closely align with the distributions of the species (then subspecies) described in Wirth & Jones (1957) (Fig. 1), and recent morphological analyses of individuals from this study supports the species level designation of these clusters^41^. For the remainder of the manuscript, we will refer to each cluster by its corresponding species name. *Culicoides occidentalis* was located in Western North America, *C. sonorensis* in the Western and Southern United States (U.S.), *C. albertensis* in the Midwest U.S. and Ontario, *C. variipennis* in the Eastern U.S. and Ontario, and a fifth genetic group suggesting the occurrence of an additional, undescribed cryptic species in San Diego, CA. Notably, eight of the 17 sites had more than one species in sympatry, and one site had three species. At four sites, seven individuals were assigned to two genetic groups with an assignment score of ~ 50% for three individuals (scores = 45, 47 and 41%) and of ~ 25% for four individuals (scores = 34, 31, 25 and 24%), which suggests the occurrence of putative F1 or other types of hybrids (e.g., F2 or backcrosses). Interestingly, these hybrids were from three different species pairings (*C. sonorensis* X *C. occidentalis*; *C. sonorensis* X *C. variipennis*; and *C. albertensis* X *C. variipennis*). These hybrid individuals also stood out in the PCA, as they segregated between their parental clusters (Fig. 2a), as well as at the base of each parental branch in the phylogenetic tree (Fig. S3). In addition to these seven hybrids, 20 individuals had a secondary assignment score between 3 and 21%, signifying potential introgression between those pairings. However, we are less confident in STRUCTURE’s ability to identify this level of ancestry as other factors can also lead to mixed assignments.

**Table 1:** Collection site information and numbers of individuals retained for the SNP analyses.

Country	State/Province	Lat	Long	Collection date	Collection method	N	Abbreviation
Canada	British Columbia	49.3065	− 119.6323	5/7/2019	Pupal rearing	5	BC
USA	California	39.0245	− 122.8515	8/14/2018	Pupal rearing	12	CACL
USA	California	38.9811	− 122.6731	8/14/2018	Pupal rearing	9	CABL
USA	California	32.5522	− 117.0628	11/7/2014	Light trap	15	CASD
USA	Idaho	43.7065	− 116.4236	8/19/2014	Light trap	14	ID
USA	Nevada	40.0521	− 118.4681	7/29/2013	Light trap	17	NV
USA	Arizona	34.5792	− 112.4258	7/21/2010	Light trap	17	AZ
USA	Utah	40.7844	− 112.1090	9/10/2018	Light trap	16	UT
USA	South Dakota	43.7438	− 101.9509	8/6/2018	Light trap	10	SD
USA	Colorado	40.6560	− 104.9878	8/8/2019	Light trap	15	COFC
USA	Colorado	39.0546	− 108.5170	7/16/2013	Light trap	7	COME
USA	Kansas	38.8793	− 98.4481	9/25/2018	Pupal rearing	16	KSLI
USA	Kansas	39.2234	− 96.5906	7/17/2018	Light trap	18	KSMA
USA	Texas	29.9515	− 99.6010	7/29/2017	Light trap	8	TX
Canada	Ontario	43.2167	− 79.9500	7/5/2013	Light trap	8	ON
USA	South Carolina	34.3080	− 81.7550	7/23/2014	Light trap	16	SC
USA	Florida	30.4782	− 84.6401	8/27/2018	Light trap	3	FL

The seven putative hybrids were excluded from the dataset used to calculate the intraspecies summary statistics (rearranged by cluster), which resulted in the isolation of 566 SNPs after more stringent filtering was applied. The mean *F*_*ST*_ between species was 0.7147 (0.6541–0.7470), roughly 9 times higher than the mean *F*_*ST*_ between the populations (i.e., localities) within each species (see below; Tables 2 & S1). Similarly, both the aR and LKC values of intra-individual genetic distance show a low level of divergence/high level of similarity within each species, including the San Diego population (Table S2). The four species-specific datasets were used to calculate the interspecies summary statistics as well as test for isolation by distance (IBD). These datasets contained 22 individuals of *C. albertensis* from four sites (3423 SNPs), 36 individuals of *C. occidentalis* from four sites (2714 SNPs), 97 individuals of *C. sonorensis* from seven sites (2357 SNPs), and 29 individuals of *C. variipennis* from four sites (2960 SNPs). The expected and observed heterozygosity, *F*_*IS*_, and number of private alleles for each species are reported in Table S2. No species-level dataset was created for the San Diego species, as this species was uncovered in a single locality.

**Table 2:** Mean pairwise *F*_*ST*_ within and between species.

Species	*C. albertensis*	*C. occidentalis*	*C. sonorensis*	*C. variipennis*
*C. albertensis*	0.055 (− 0.009 to 0.116)	–	–	–
*C. occidentalis*	0.707	0.411 (0.143–0.704)	–	–
*C. sonorensis*	0.709	0.730	0.029 (0.006–0.069)	–
*C. variipennis*	0.654	0.747	0.730	0.026 (− 0.006 to 0.045)
San Diego pop	0.714	0.719	0.706	0.734

The between species *F*_*ST*_ values (below diagonal) were calculated using 566 SNPs and the within-species values (on diagonal) is the mean *F*_*ST*_ calculated from individual species-specific datasets (see Table S4).

**Figure 3:**
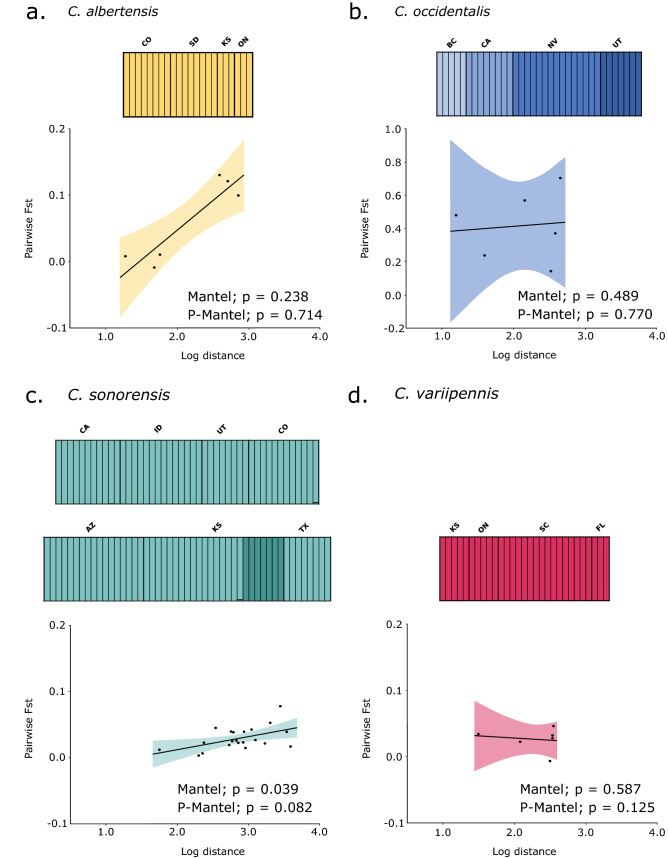
For each species, an independent SNP dataset was used to calculate the most suitable K using fastSTRUCTURE with the inferred clusters denoted by varying shades. A Mantel and partial Mantel (P-Mantel) test was used to test for IBD (shown as pairwise *F*_*ST*_ by log geographic distance) for each species in Genepop. The individuals from San Diego, CA are not included here as they were only found in a single population.

When examining each species individually, *C. albertensis,* had no evidence of population structure (K = 1), and had low genetic differentiation among locations (mean *F*_*ST*_ = 0.054) (Fig. 3a; Table 2). Although there does seem to be a pattern of IBD, this was found to not be significant in this species (Mantel test, *P* = 0.238; Partial Mantel test, *P* = 0.714). The low number of locations sampled potentially limits the statistical power of these correlations. The results obtained for *C*. *occidentalis* showed much more divergence compared to the other species, with populations being strongly differentiated from each other (mean *F*_*ST*_ = 0.411) (Table 2). Additionally, the fastSTRUCTURE analysis suggested that each location of *C. occidentalis* sampled is distinct (K = 4) (Fig. 3b). While no IBD was found (Mantel test, *P* = 0.489; Partial Mantel test, *P* = 0.770), there seems to be a considerable amount of geographic isolation among populations of this species, with pairwise *F*_*ST*_ values ranging from 0.14 to 0.70 (Table S4). Additionally, significant levels of dissimilarity were found between individuals from California and those from the other three populations (Fig. S4). In contrast, low genetic differentiation among locations were found for *C. sonorensis* (mean *F*_*ST*_ = 0.029), with varying levels of support for IBD in this species (Mantel test, *P* = 0.039; Partial Mantel test, *P* = 0.082) (Fig. 3c; Table 2). For this reason, the individuals from Colorado were combined into a single location, as were the individuals from Kansas. The fastSTRUCTURE analysis suggested the occurrence of population structure in *C. sonorensis* (K = 2), with some individuals from Kansas belonging to a distinct group, though these were not highly divergent from any other *C. sonorensis* location (Table S4). Individuals of *C*. *variipennis* exhibited no evidence of population structure (K = 1) or of IBD (Mantel test, *P* = 0.587; Partial Mantel test, *P* = 0.125) (Fig. 3d). Consistently, almost no genetic differentiation was found among locations of this species (mean *F*_*ST*_ = 0.026) (Table 2).

### Haplotype network

**Figure 4:**
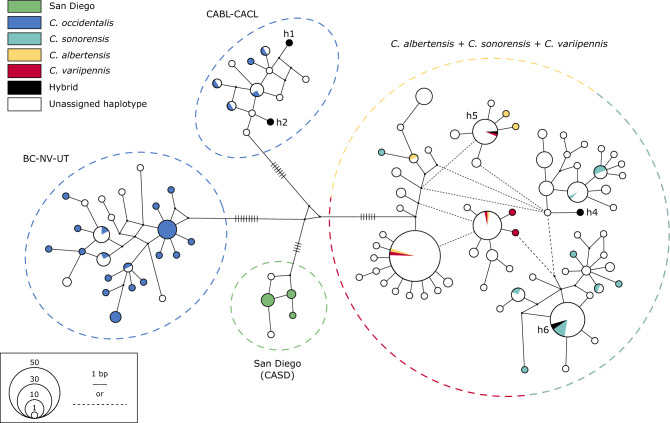
A haplotype network inferred by a median-joining method, using 285 mitochondrial (mt) DNA sequences of the *C. variipennis* complex from 27 states in the U.S., as well as British Columbia and Ontario, Canada. The size of each circle represents the frequencies of the haplotype and the length of the lines connecting the circles corresponds to number of bp differences. Note that the dotted black lines also represent a single bp change. The 67 sequences obtained in the present study (see Fig. 1) are colored according the clusters assigned from the structure analysis. The four main groups of haplotypes (see Results) are circled.

In total, 285 midges were included in the analysis of a 546 bp region of the COI gene. Four distinct haplogroups were identified with substantial genetic divergence between groups (*p-*distance = 2.99–3.30%) and little divergence within groups (*p*-distance = 0.25–0.86%; Fig. 4; Table 3). Consistent with the SNP datasets, *C. occidentalis* formed a divergent haplogroup, separated from the rest of its range. The mean percent divergence between the two *C. occidentalis* groups (2.99%) was similar to its divergence from the other species (3.01–3.30%). The San Diego population also clustered as a distinct group, with a similar level of divergence from the other species (3.01–3.03%). Interestingly, *C. albertensis*, *C. sonorensis*, and *C. variipennis* were not separated from each other, and in some cases, *C. albertensis* and *C. variipennis* shared identical haplotypes (Fig. 4, S5). Furthermore, these three species exhibit a mean percent divergence between individuals (0.80%) similar to the divergence observed among individuals within *C. occidentalis* (Table 3)*.* Other than the grouping of *C. occidentalis* in California, there was no geographic clustering observed.

**Table 3:** Mean percent divergence (p-distance) within and between species clusters based on the COI gene (ranges listed in parentheses).

Clade	occ (CABL)	occ (BC-NV-UT)	San Diego pop	alb-son-var
occ (CABL)	0.48 (0.00*–*0.73)	–	–	–
occ (BC-NV-UT)	3.99 (3.20*–*5.49)	0.86 (0.00*–*1.65)	–	–
San Diego pop	3.01 (2.38*–*4.21)	3.66 (2.75*–*4.76)	0.25 (0.00*–*0.66)	–
alb-son-var	3.30 (2.75*–*5.12)	3.76 (3.30*–*6.04)	3.03 (2.38*–*4.21)	0.80 (0.00*–*2.74)

Based on overall similarity, *C. occidentalis* was split into two groups (CABL; and BC-NV-UT) and *C. albertensis*, *C. sonorensis*, and *C. variipennis* were grouped into a single clade (alb-son-var).

## Discussion

Our study provides valuable insights into the population genetics of the *C*. *variipennis* species complex and highlights the presence of potential cryptic species. For most of the species examined, minimal genetic divergence was observed across locations, suggesting the maintenance of gene flow even over large geographic distances. The only exception was *C. occidentalis*, which showed a high level of geographic isolation, as well as two distinct COI haplogroups. We confirmed that mitochondrial data is not reliable to differentiate three out of the five species, due to the lack of segregation between the mitochondrial haplotypes of *C. albertensis*, *C. sonorensis*, and *C. variipennis*. This stands in stark contrast to their clear differentiation and high level of divergence inferred from the SNP data. Though a substantial amount of divergence exists between all five species, low levels of hybridization, and potentially introgression, are present in sympatric populations. While we do not know the fitness of these hybrids, but this could suggest that strong post-zygotic isolation barriers may have yet to evolve in this group. Thus, pre-zygotic isolation through either ecological or behavioral segregation is a possible mechanism maintaining divergence within this complex. With a considerable amount of geographic overlap between some species (Fig. 1), each sympatric population is potentially experiencing a set of unique selective pressures to maintain species boundaries.

The high degree of genetic differentiation between clusters inferred by the SNP data supports the current species groupings of the *C. variipennis* complex (*C. occidentalis*, *C. sonorensis*, and *C. variipennis*), as well as raising *C. albertensis* and a cryptic species in San Diego, California to species status (Fig. S5). While this putative new species was only collected in San Diego, its true distribution could extend well into Mexico. While clear divergence was observed in the SNP data, the mitochondrial data showed a different pattern of divergence. *Culicoides albertensis*, *C. sonorensis*, and *C. variipennis* have a considerable amount of genome-wide differentiation (Fig. 1); however, there was no clear differentiation of the COI gene (Fig. 4). In fact, several individuals of *C. albertensis* and *C. variipennis* shared identical haplotypes. Multiple studies have shown a high degree of genetic similarity in mtDNA between *C. sonorensis* and *C. variipennis*^23,24,42^, though it was proposed that this was due to misidentifications. As all of the individuals included in our mitochondrial haplotype analysis from the current study were identified to species using the SNP data, this lack of mitochondrial separation must have an underlying biological cause. This finding can result from historical introgression with “leaky” pre-zygotic isolation, or semipermeable species boundaries, which have been shown to produce mitochondrial introgression without detectable nuclear DNA introgression in some taxa^43,44^. This is likely due to the fact that the mitochondrial genome is independent of the nuclear genome and thus unlinked to the genes contributing to reproductive isolation^45^. However, we cannot rule out that other possibilities could have caused this discordance, such as recent speciation and incomplete lineage sorting or selection^13,46^. Regardless, it appears that the evolution of the mitochondrial genome is not congruent with the species tree of the *C. variipennis* complex. Notably, the SNP phylogenetic tree shows that *C. occidentalis*, and not *C. sonorensis*, is the sister taxa of *C. albertensis* and *C. variipennis* (Fig. S5). This suggests that the mtDNA similarity between *C. albertensis*, *C. sonorensis* and *C. variipennis* could stem from ongoing hybridization and introgression, rather than incomplete lineage sorting.

Little to no IBD or structure was found within *C. albertensis*, *C. sonorensis*, and *C. variipennis* indicating a substantial amount of connectivity among localities of these species (Fig. 3a,c,d). The number of populations inferred by fastSTRUCTURE for *C. sonorensis* was K = 2; however, a mean pairwise *F*_*ST*_ of 0.0287 suggests that a high amount of gene flow exists between all locations. This could be an artifact of the propensity of delta K inferring two populations^47^ or from a high level of relatedness among individuals from KS (Fig. S6). Interestingly, although no IBD was found in *C. occidentalis*, each location of this species clustered as a distinct population (Fig. 3b). The lack of IBD is therefore not indicative of a single, genetically homogeneous population, but rather stems from high levels of divergence between populations regardless of their geographic distances. Focusing sampling efforts on each of these species will surely permit robust landscape genetic approaches to gain understanding of the evolutionary forces driving population structure in this group. This will allow studying whether *Culicoides* population structure is characterized by uniform or discontinuous isolation by distance, as well as, isolation by adaptation (IBA) or by environment (IBE)^48^.

The strong genetic divergence between the *C. occidentalis* from California and the other populations was observed in both the SNP and mtDNA data (Tables 2, 3, Fig. 4). It is possible that this may represent a further cryptic species with a dispersal barrier created by the Sierra-Nevada mountain range (Fig. S4). This high level of differentiation within *C. occidentalis* could be due to geographic isolation alone; however, endosymbionts have also been shown to significantly increase mitochondrial diversity in the presence of geographic structure^49,50^. Naturally occurring endosymbionts have been found in *Culicoides* midges, including *C. sonorensis*^51,52^, and recently, a *Cardinium* sp. was linked to mitochondrial divergence in *C*. *imicola*^53^. Further screening is needed to determine the diversity and abundance of endosymbionts infecting *Culicoides* midges, though the possibility remains that they could be playing a role in the phylogeographical structure of *C. occidentalis* if they are causing incompatibility between populations. Additionally, patchiness of the specialized larval habitat of *C. occidentalis*, not present in the other members of the *C. variipennis* complex, could create isolation between populations, as well as reduce the number of individuals within each population. A small effective population size with little to no immigration would allow for a strong effect from drift^54^. While the populations of *C. occidentalis* outside of California were less diverged from one another, the lowest pairwise *F*_*ST*_ values between these populations were still greater than the highest pairwise values observed within any other species, consistent with the findings of Holbrook et al. (2000) (Table 2).

Similar to other species of *Culicoides*^30,32,33,55^*,* high values of the inbreeding coefficient (*F*_*IS*_) were observed in all species investigated in this study (Table S2). Although these previous studies have suggested that the observed high *F*_*IS*_ are an artifact from a large number of null alleles, the consistent reporting of these findings across various species using several types of molecular markers lends support to the hypothesis that high inbreeding has a biological origin in this genus. High levels of inbreeding and heterozygote deficiencies are common among mosquitoes^56–58^, even when using markers with a low level of null alleles^59,60^. Goubert et al. (2016) considered the typical *Aedes albopictus* population as “a network of interconnected breeding sites, each with a high level of inbreeding”. Although we cannot rule out all other possibilities, our results strongly suggest that some aspects of the reproductive biology of *Culicoides* induce inbreeding within populations. High *F*_IS_ and low *F*_ST_ between populations can stem from high levels of migration between populations (i.e., homogenizing allele frequencies at large scale), followed by matings with close relatives within populations (i.e., increasing homozygosity without altering allelic frequencies). It is also possible that our sampling approaches (single night trapping) led to capturing cohorts of closely related individuals.

Low levels of hybridization were found in some sympatric populations involving several different species pairings. Under laboratory conditions, mating between *C. sonorensis* and *C. occidentalis* can produce viable offspring for at least six generations, though the hatch rate of the progeny is dependent on the species of the mother^25^. A cross of female *C. sonorensis* and male C*. occidentalis* only yields a 7% hatch rate whereas the reciprocal cross yields a 75% hatch rate. This asymmetrical hybrid viability is likely caused by cytonuclear incompatibility^61,62^, though endosymbionts have also been shown to cause reproductive incompatibility^63^. Upon secondary contact of closely related species, and in the absence of post-zygotic reproductive isolation, the production of unfit hybrids can induce the rapid evolution of premating barriers^2,64–66^. In most populations however, *C. sonorensis* females are unlikely to come across *C. occidentalis* males due to differences in mating behavior. Conversely, *C. occidentalis* females do come into contact with *C. sonorensis* males, who do not appear to have mate discrimination^67^, and will likely attempt to mate with these heterospecific females. As there are demographic disparities (population size and structure) between these two species, as well as viable offspring produced from this cross, rampant hybridization and asymmetric introgression would be detrimental to *C. occidentalis*^68^. Strong selection against hybridization can maintain species boundaries, but as two of the ten *C. occidentalis* collected from Borax Lake in California (CABL) appeared to be F1 hybrids (Fig. 1), another mechanism, potentially differences in the larval habitat or mating behavior, appears to be limiting directional introgression from *C. sonorensis*.

*Culicoides occidentalis* females lay their eggs in highly saline environments (up to 88.0 parts per thousand (ppt))^69^, whereas *C. sonorensis* eggs will not hatch in water with salinity over 20.0 ppt^70^. However, ecological exclusion via the larval habitat would only limit introgression if the hybrids were inviable in highly saline environments, which does not appear to be the case. The difference in mating behavior between these two species may be a more likely mechanism by which the detrimental effects of hybridization are diminished. *Culicoides occidentalis* mates at the larval habitat while *C. sonorensis* mates at or near a host^22,71^. Even if a *C. occidentalis* female mated with a *C. sonorensis* male, she would return to the high saline pools to lay her eggs and these hybrid offspring would have a high chance of only backcrossing within the *C. occidentalis* lineage. While only two *C. occidentalis* x *C. sonorensis* hybrids were tested in this study, both had *C. occidentalis* mothers (Fig. 4), providing evidence that this scenario takes place in nature. However, this type of isolation would not explain how *C. sonorensis* and *C. variipennis* maintain species boundaries in sympatry as they share a larval habitat. Further studies are needed to determine the mechanisms behind reproductive isolation within this group.

The *C*. *variipennis* complex is one of many vector groups in which species delimitation can be challenging^46,72–76^; however, species identification is an integral part of vector surveillance. The species status of these group members has implications for vector surveillance, as any ambiguity in identification will lead to unreliable data. For example, while *C. albertensis* and *C. sonorensis* occur in sympatry, only *C. sonorensis* is reported as a vector species^77^. The addition of the non-vector species when conducting serological surveys could lead to a severe underestimation of the infection rate within the vector species. As BTV and EHDV are expanding northward into eastern Canada^78^, it has been suggested that the dispersal of *C. sonorensis* into new areas could be to blame for this incursion^42^. Specimens assigned to *C. sonorensis* by Jewiss-Gaines et al. (2017) were included in the present study and cluster instead with *C. albertensis* (“ON”, Fig. 1). Thus, there are likely alternative reasons for the range expansion of these viruses, including an unidentified vector species outside of the *C. variipennis* complex, such as *C. stellifer*^79,80^. Molecular tools for accurate species-level delimitation within this complex is sorely needed for proper vector surveillance. Additionally, the detection of hybridization between a non-vector and vector species may be evidence of recent speciation, but it also highlights a potential path of introgression for genes controlling vector competency^81,82^.

Our study shows that using a population genomic approach to analyze sibling species can identify species-level divergence, fine-scale genetic structuring within species, and uncover the existence of hybrids and cryptic species in *Culicoides*. Radiation within the *C. variipennis* complex occurred despite the long-range dispersal capabilities of biting midges as well as hybridization between sympatric species. This does not preclude historical geographic isolation; however, we believe that behavioral and ecological isolation may have shaped evolution within this group or is at least maintaining the current species boundaries. Significant geographic isolation was only found between populations of *C. occidentalis*, but more sampling is needed to determine if the lack of gene flow between California and the other populations represents an incipient speciation event or IBD. Additionally, focusing efforts in these various hybrid zones may provide a better understanding of the evolution of reproductive isolation in this group. Cryptic species have been reported in a number of other *Culicoides* species complexes^83^ and the analyses presented here could help to identify these putative species. Delimiting the species in these complexes, will not only aid in vector surveillance efforts, but continued study of the speciation of closely related vector and non-vector species could produce valuable evolutionary insights into vector competency.

## Materials and methods

### Sample collection and sequencing

*Culicoides* midges were collected from 17 sites across the United States and Canada (Table 1). Specimens were collected either as pupae and reared to adulthood, or as adults using CDC light traps baited with CO_2_ and UV light (Bioquip 2836BQ). Individuals morphologically assigned to the *C*. *variipennis* complex were sorted out from the by-catch and stored in 95% ethanol at -80 °C. Total DNA was extracted from individuals using a Puregene extraction protocol (Gentra Systems, Inc., D-5500A) with the addition of glycogen (ThermoFisher, R0561) to increase yields. DNA was only extracted only from females as their larger body size (compared to the males) produced sufficient amount of DNA for next-gen sequencing. The DNA quality was checked using gel electrophoresis and DNA concentration was measured using a Qubit 3.0 fluorometer and a Qubit dsDNA HS assay kit (Invitrogen, Q33230). A total of 300–400 ng of DNA per sample was sent to Floragenex, Inc. for library preparation using the protocol from Truong et al. (2012). DNA was digested using the restriction enzymes *MseI* and *PstI*. After PCR amplification, the samples in each plate were pooled and sequenced on a lane of single-end 100 bp sequencing on a HiSeq4000 at the University of Oregon Genomics Facility, Eugene, OR.

### Raw sequence filtering and processing

Raw sequence quality was first assessed using FastQC v.0.11.9 and MultiQC v.1.7^84,85^, and then reads were filtered and processed using Stacks v.2.3^86^. Reads with a phred score below 25 were removed as well as individuals with a > 75.0% missing data. Next, reads were aligned to the *C. sonorensis* genome^87^ (Accession: PRJEB19938) using the Burrows-Wheeler Aligner (BWA-mem)^88^. Finally, aligned reads were run through the reference-based pipeline of Stacks. Filtering options were set to only include loci found in at least half of the sampling locations (-p 8) and those occurring in at least 50% of individuals within those sites (-r 0.5)^89^. The minimum allele frequency was set to 0.05 to protect against potential sequencing errors^90^, and only the first SNP per locus was kept to minimize linkage disequilibrium between SNPs from influencing population structure and phylogenetic analyses. All subsequent file reformatting was done with PGDSpider v.2.1.1.5^91^.

### Clustering analysis

Population structure in the overall dataset was evaluated using fastSTRUCTURE v.1.04, with Structure_threader utilized to parallelize distinct runs of K^92,93^. Models were fitted with the number of genetic clusters (K) set to range from 1 to 10. The most suitable value of K was selected using the *chooseK.py* function from the fastSTRUCTURE package which selects the model that maximizes the marginal likelihood of the data. Using the output from fastSTRUCTURE and Distruct v.2.3 (http://distruct2.popgen.org), a bar plot was created where each individual is represented by a vertical line divided into K colored segments with the length of each segment being proportional to the estimated membership in each of the inferred K groups. A map of the structuring at each collection site was created using Inkscap v.1.1 (https://inkscape.org/). The clustering of individuals into the distinct genetic groups was also visualized using a principal component analysis (PCA) and a discriminant analysis of principal components (DAPC). The most likely number of genetic groups was inferred by the *find.clusters* algorithm for the PCA and the optimal number of principal components to inform the DAPC was defined using the function *optim.a.score*. Both were performed in R^94^ through the *adegenet* package^95^.

Any individual with more than 25% of their loci grouping with a second cluster in the fastSTRUCTURE analysis was marked as a hybrid and removed from the phylogenetic analysis, due to the uncertainty of assigning them to a given species. Maximum likelihood phylogeny among individuals was run using RAxML v.8.2.12^96^. An acquisition bias correction was applied to the likelihood calculations as alignments were solely composed of SNPs, with each invariant site removed through Phrynomics (https://github.com/bbanbury/phrynomics)^97^. The GTR + G nucleotide substitution model was used for each search. A rapid bootstrap analysis and search for the best-scoring maximum likelihood tree was executed using the extended majority rule-based bootstopping criterion to achieve a sufficient number of bootstrap replicates^98^. Additionally, to cross-validate our results, a second phylogeny was inferred in W-IQ-Tree version 1.6.12^99^, using the TVM + F + G4 substitution model determined by ModelFinder^100,101^. Branch support was calculated using 1000 ultrafast bootstraps^102^ and a Shimodaira–Hasegawa like approximate likelihood-ratio test (SH-aRLT)^102,103^.

To measure the amount of divergence between genetic clusters, a new SNP dataset was generated with individuals grouped by cluster rather than locality. Additionally, to measure the amount of divergence within each genetic cluster, four cluster-specific datasets (grouped by site) were also generated. SNPs were obtained from these new datasets using the same processing methods above except with more stringent filtering parameters. Only SNPs that occurred in at least 75% of the clusters or collection sites and at least half of the individuals within those groups were included. Genetic diversity estimates (*F*_*IS*_, *H*_*E*_, and *H*_*O*_) and population differentiation (pairwise *F*_*ST*_) were calculated for each species dataset using Genepop v.4.7.0^104^. Population differentiation was not calculated for the new species found in San Diego, as only a single population of this species was uncovered. Rousset’s distance aR^105^ and Loiselle’s kinship coefficient (LKC)^106^ were calculated respectively with SPAGeDi v.1.5^107^. Geographic distances among localities were calculated as both Euclidean and anisometric distances and a Mantel test and a Partial Mantel test were preformed to test for isolation-by-distance (IBD)^108^. Tests for areas of significant genetic dissimilarity among individuals using aR were implemented in MAPI using 1000 replications^109^.

### Mitochondrial sequencing and haplotype network

Mitochondrial DNA haplotypes were obtained from a subset of 67 individuals from the five genetic clusters. PCR reactions were performed using a Taq-Pro COMPLETE kit (Denville Scientific, CB4065-4) targeting a partial region of the COI gene with the Lep50 primer set from Folmer et al. (1994) and the thermocycler profile from Herbert et al. (2003). PCR products were cleaned using an EXOSAP-IT kit (ThermoFisher, 78201.1.ML) and prepared for sequencing using a BigDye Terminator v.3.1 Cycle Sequencer Kit (Applied Biosystems, 4337454). Sanger sequencing was done using an Applied Biosystems 3500 Genetic Analyzer. Chromatograms were cleaned and aligned using the software Geneious v.9.1^110^.

A haplotype network analysis was conducted using the 67 COI sequences obtained in this study combined with 218 *C. variipennis* complex sequences previously collected from 25 states across the U.S.^111^. Sequences were aligned in MEGA v.10.1.8^112^ and trimmed to ensure all sequences contained identical lengths. A median-joining analysis was performed using NETWORK v.5.0.1.0^113^. Specimens collected in this study were assigned a color based on the results from the SNP clustering analyses while the remaining samples were left unassigned. All individuals were used to calculate the mean uncorrected *p-*divergence between and within the different groupings inferred from the haplotype network using MEGA.

## Supplementary Information

Supplementary Information.

## Data Availability

The data reported in this study will be deposited in the Open Science Framework database upon acceptance, https://osf.io (10.17605/OSF.IO/E3Z72). Mitochondrial sequences obtained in the current study have been deposited under Genbank Accession Numbers OL604713—OL604779.
