# Supplementary material for: Species delimitation and mitonuclear discordance within a species complex of biting midges
Source: Sci Rep. 2022 Feb 2;12:1730. doi: 10.1038/s41598-022-05856-x (PMC8810881; doi:10.1038/s41598-022-05856-x)
Supplement: Supplementary file 1 — Supplementary Information. [file 41598_2022_5856_MOESM1_ESM.pdf]

Supplemental Table 1. Genotypic frequencies for each sample were compared using a log-likelihood (G)-based test of differentiation using Genepop version 4.7.0. Pairwise F_{ST} values between populations are listed below the diagonal and those found to be statistically similar are bolded ($p > 0.05$). Populations are listed as: species abbreviation_collecting site (Table 1). alb = *C. albertensis*, occ = *C. occidentalis*, son = *C. sonorensis*, var = C. variipennis.

sp_pop	alb_COFC	alb_KSMA	alb_ON	alb_SD	occ_BC	occ_CACL	occ_NV	occ_UT	son_AZ	son_CACL	son_CO	son_ID	son_KSMA	son_TX	son_UT	var_FL	var_KSMA	var_ON	var_SC
alb_COFC	-																		
alb_KSMA	**0.0233**	-																	
alb_ON	**0.1240**	**0.2119**	-																
alb_SD	**0.0143**	**0.0412**	**0.1926**	-															
occ_BC	0.7114	0.7871	0.7222	0.7702	-														
occ_CACL	0.7004	0.7614	0.7021	0.7536	0.3146	-													
occ_NV	0.7268	0.7674	0.7253	0.7642	**0.1091**	0.2511	-												
occ_UT	0.7132	0.7733	0.7210	0.7654	0.2770	0.3021	0.1827	-											
son_AZ	0.7073	0.7378	0.7050	0.7408	0.7521	0.7471	0.7649	0.7592	-										
son_CACL	0.6727	0.7131	0.6641	0.7250	0.7311	0.7254	0.7482	0.7406	**0.0356**	-									
son_CO	0.6835	0.7155	0.6793	0.7243	0.7382	0.7312	0.7553	0.7462	**-0.0002**	**0.0268**	-								
son_ID	0.6898	0.7274	0.6870	0.7342	0.7446	0.7357	0.7578	0.7507	**-0.0002**	**0.0107**	**0.0011**	-							
son_KSMA	0.7057	0.7325	0.7045	0.7362	0.7514	0.7440	0.7636	0.7575	**0.0173**	**0.0647**	**0.0244**	**0.0300**	-						
son_TX	0.6966	0.7522	0.6968	0.7532	0.7770	0.7638	0.7796	0.7766	**0.0153**	**0.0212**	**0.0220**	**0.0011**	**0.0297**	-					
son_UT	0.6895	0.7309	0.6876	0.7387	0.7461	0.7396	0.7605	0.7528	**0.0140**	**0.0504**	**0.0230**	**0.0078**	**0.0368**	**0.0180**	-				
var_FL	0.6222	0.7103	**0.5464**	0.6971	0.7828	0.7489	0.7657	0.7710	0.7292	0.6958	0.7060	0.7167	0.7282	0.7341	0.7204	-			
var_KSMA	0.6256	0.6965	0.5586	0.6841	0.7783	0.7541	0.7707	0.7708	0.7431	0.7143	0.7200	0.7332	0.7397	0.7533	0.7374	**0.0759**	-		
var_ON	0.6116	0.6729	0.5269	0.6723	0.7632	0.7406	0.7645	0.7602	0.7269	0.6907	0.7016	0.7131	0.7249	0.7270	0.7166	**0.0136**	**0.0146**	-	
var_SC	0.6786	0.7206	0.6116	0.7103	0.7868	0.7681	0.7826	0.7832	0.7595	0.7423	0.7444	0.7555	0.7553	0.7722	0.7593	**0.0826**	**0.0311**	**0.0255**	-
CASD	0.7185	0.7548	0.7164	0.7539	0.7550	0.7444	0.7527	0.7549	0.7324	0.7158	0.7220	0.7284	0.7310	0.7461	0.7324	0.7414	0.7348	0.7294	0.7500

Supplemental Table 2. Comparison of pairwise genetic distances within and between species. Rousset's distance (aR) measures the genetic differentiation among individuals rather than the differentiation between populations (pairwise Fst), and Loiselle's kinship coefficient (LKC) is a measure of genetic dissimilarity.

Pairwise Rousset's distance between individuals (aR; 'a' in Rousset, 2000)

Species	*C. albertensis*	*C. occidentalis*	*C. sonorensis*	*C. variipennis*	CASD
C. albertensis	0.8966	-	-	-	-
C. occidentalis	5.0278	0.8129	-	-	-
C. sonorensis	5.1911	5.6082	0.8405	-	-
C. variipennis	3.8555	5.4976	5.4020	0.5080	-
**CASD**	4.8716	4.8380	4.9001	4.7081	0.4706

Pairwise KINSHIP coefficients (LKC)

Species	*C. albertensis*	*C. occidentalis*	*C. sonorensis*	*C. variipennis*	*CASD*
C. albertensis	0.8122	-	-	-	-
C. occidentalis	-0.0014	0.8800	-	-	-
C. sonorensis	-0.2800	-0.3349	0.3594	-	-
C. variipennis	0.2058	-0.0934	-0.3243	0.8754	-
**CASD**	0.0227	0.0544	-0.2106	0.0492	0.9255

Pairwise Fst (Table 2)

Species	*C. albertensis*	*C. occidentalis*	*C. sonorensis*	*C. variipennis*	*CASD*
C. albertensis	0.0550	-	-	-	-
C. occidentalis	0.7070	0.4110	-	-	-
C. sonorensis	0.7090	0.7300	0.0290	-	-
C. variipennis	0.6540	0.7470	0.7300	0.0260	-
**CASD**	0.7140	0.7190	0.7060	0.7340	-

Supplemental Table 3. Genetic diversity based on SNP data for all species and within collection sites by species. The inbreeding coefficient (F_{IS}), expected heterozygosity (H_e), and observed heterozygosity (H_o) were calculated in Genepop v.4.7.0. Private alleles were identified using populations v.2.41.

**Species**	**N**	F_{IS}	H_e	H_o	Private Alleles
C. albertensis	22	0.4240	0.1020	0.0587	11
C. occidentalis	36	0.4989	0.0936	0.0469	28
C. sonorensis	94	0.4254	0.0973	0.0559	56
C. variipennis	29	0.3084	0.0850	0.0588	13
San Diego pop	15	0.3719	0.0790	0.0496	31

C. albertensis

Populations	**N**	F_{IS}	H_e	H_o
CO	8	0.5914	0.2868	0.1172
KS	3	0.3294	0.2488	0.1669
ON	3	0.4638	0.2851	0.1529
SD	8	0.4748	0.2720	0.1429

C. occidentalis

Populations	**N**	F_{IS}	H_e	H_o
BC	5	0.3344	0.2007	0.1336
CA	8	0.6018	0.1891	0.0753
NV	15	0.3935	0.2401	0.1456
UT	7	0.3929	0.2250	0.1366

C. sonorensis

Populations	**N**	F_{IS}	H_e	H_o
AZ	17	0.4141	0.2701	0.1583
CA	11	0.6839	0.2559	0.0809
CO	13	0.4465	0.2776	0.1536
ID	14	0.6567	0.2503	0.0859
KS	23	0.4036	0.2710	0.1616
TX	8	0.7639	0.2474	0.0584
UT	8	0.4493	0.2686	0.1479

C. variipennis

Populations	**N**	F_{IS}	H_e	H_o
FL	3	0.6149	0.2739	0.1055
KS	5	0.3311	0.2767	0.1851
ON	5	0.4780	0.2862	0.1494
SC	16	0.3418	0.2776	0.1827

Supplemental Table 4. Pairwise F_{ST} values between populations of each species estimated in Genepop v.4.7.0. Population abbreviations can be found in Table 1.

C. albertensis

Populations	CO	KS	ON
CO	-	-	-
KS	-0.0085	-	-
ON	0.0912	0.1161	-
SD	0.0082	0.0110	0.1089

C. occidentalis

Populations	BC	CA	NV
BC	-	-	-
CA	0.7044	-	-
NV	0.1430	0.4830	-
UT	0.3723	0.5689	0.2365

C. sonorensis

Populations	AZ	CA	CO	ID	KS	TX
AZ	-	-	-	-	-	-
CA	0.0368	-	-	-	-	-
CO	0.0062	0.0393	-	-	-	-
ID	0.0230	0.0213	0.0166	-	-	-
KS	0.0264	0.0686	0.0254	0.0500	-	-
TX	0.0267	0.0395	0.0247	0.0183	0.0430	-
UT	0.0065	0.0362	0.0138	0.0192	0.0365	0.0250

C. variipennis

Populations	FL	KS	ON
FL	-	-	-
KS	0.0445	-	-
ON	-0.0059	0.0317	-
SC	0.0340	0.0276	0.0231

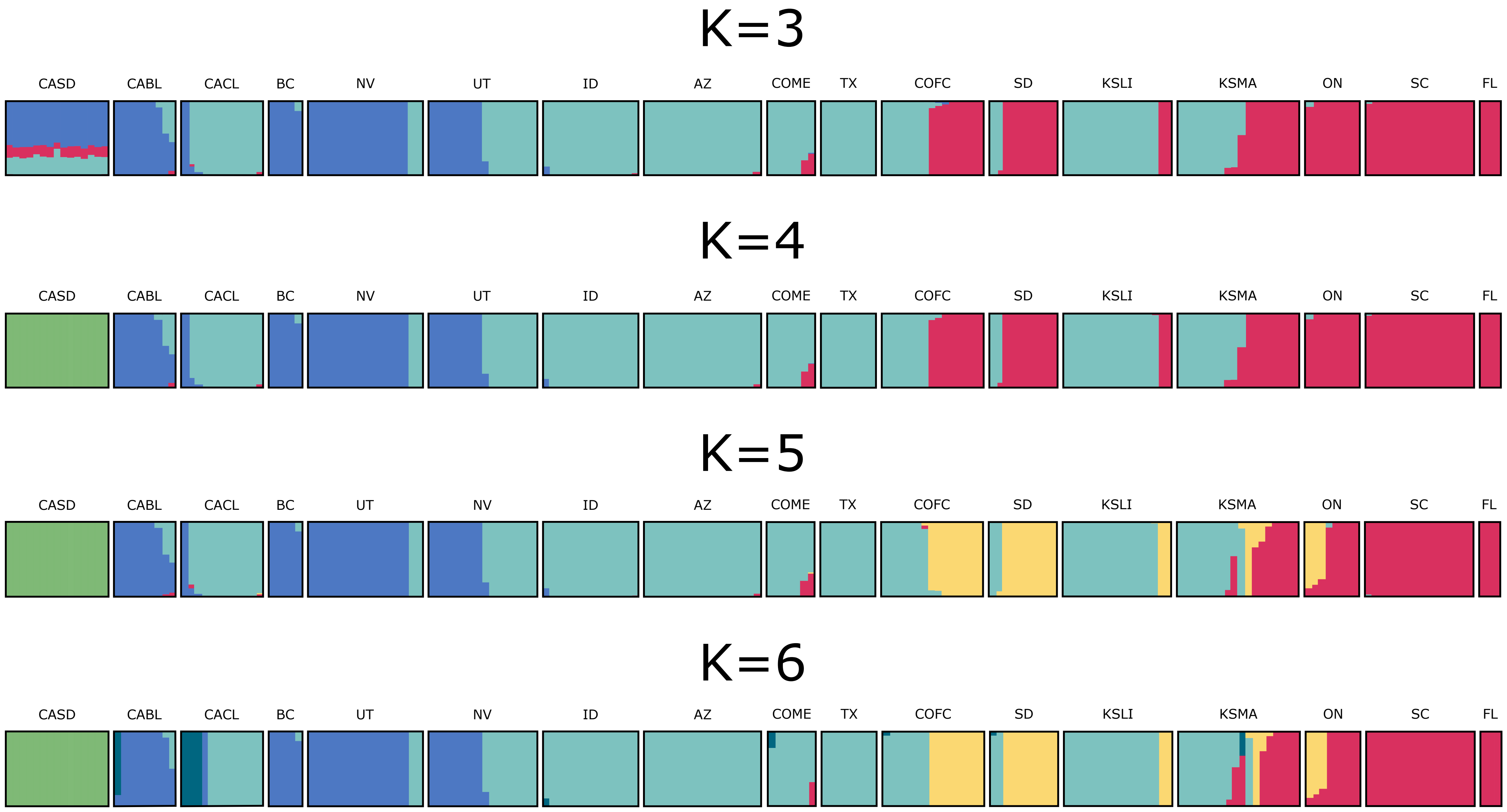

Supplemental Figure 1. fastSTRUCTURE results assuming four, five, and six population clusters (K= 3, K=4, K=5, and K=6). Each column represents an individual and are grouped by collection site with each color representing a cluster.

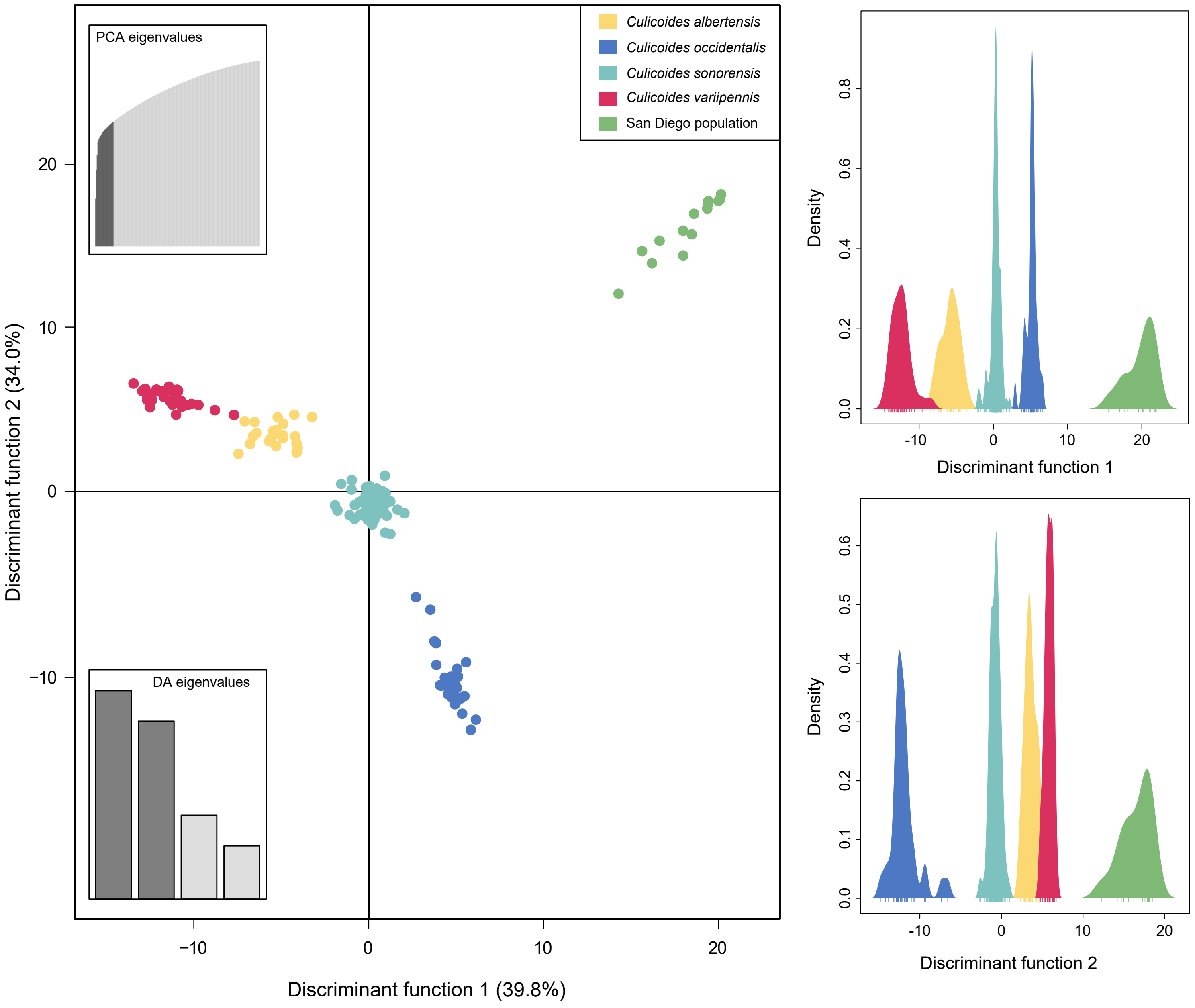

Supplemental Figure 2. Individuals are grouped according the Discriminant Analysis of Principal Components (DAPC) with the best support for K = 5 genetic clusters. The DAPC was performed in R using the adegenet package.

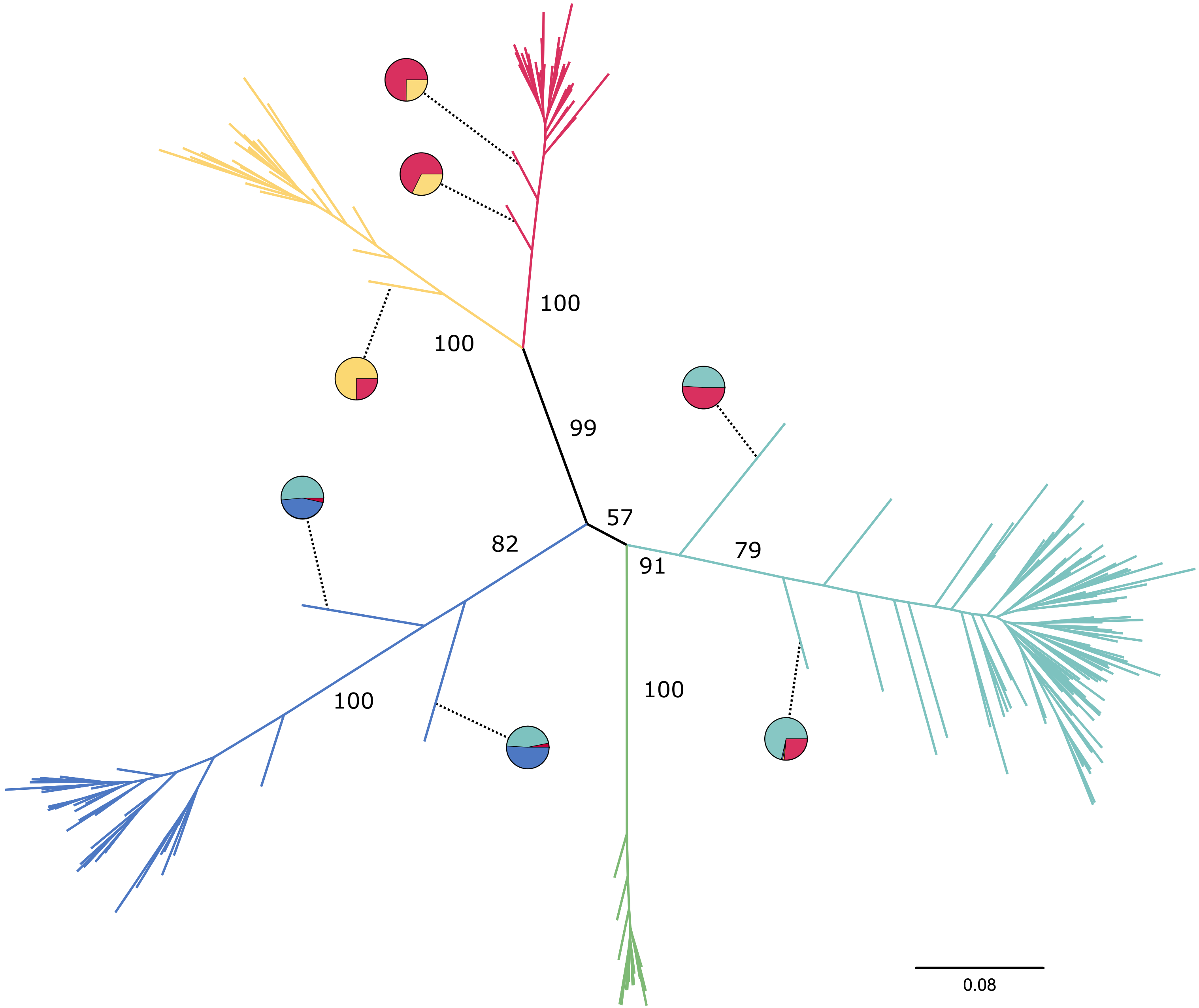

Supplemental Figure 3. Unrooted phylogenetic tree based on 206 individuals inferred from 3612 SNPs with the putative hybrids represented. Clade colors represent the putative species inferred from the structure analysis. Bootstrap support values are based on 500 rounds of bootstrapping using RAxML's rapid bootstrapping algorithm, and are shown only if ≥50% and are not shown within species groups.

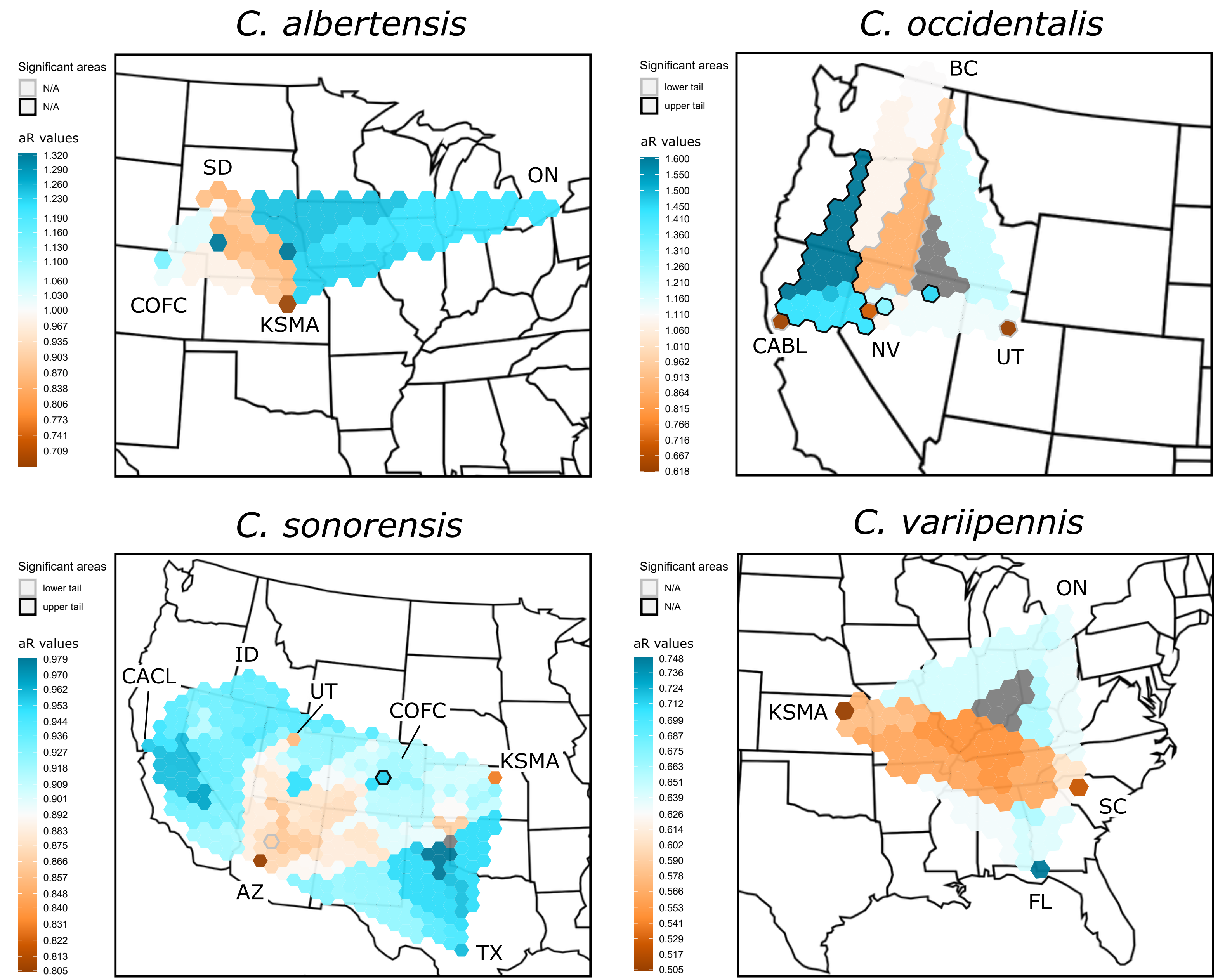

Supplemental Figure 4. The spatial variation in the degree of dissimilarity between individuals were mappped for each species using MAPI. Areas of significantly higher and lower levels of gentic dissimilarity are outlined in black and grey respectivley.

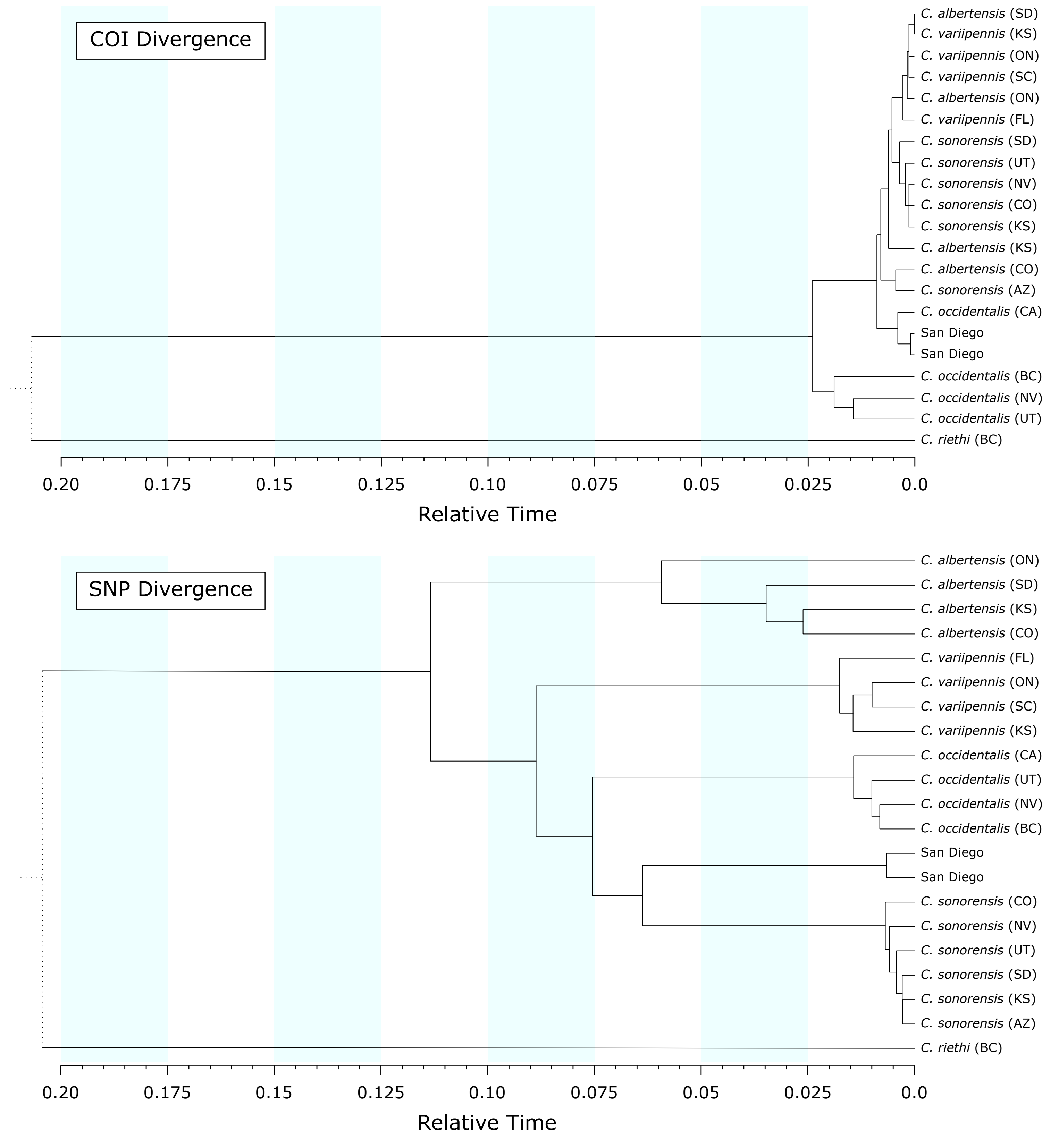

Supplemental Figure 5. Time trees for the COI gene and SNP data created using the RealTime method in MEGA. A subset of each species were selecteed for these analyses with *C. riethi* as the outgroup. Divergence times were estimated using a maximum liklihood method under a GTR+G+I substitution model.

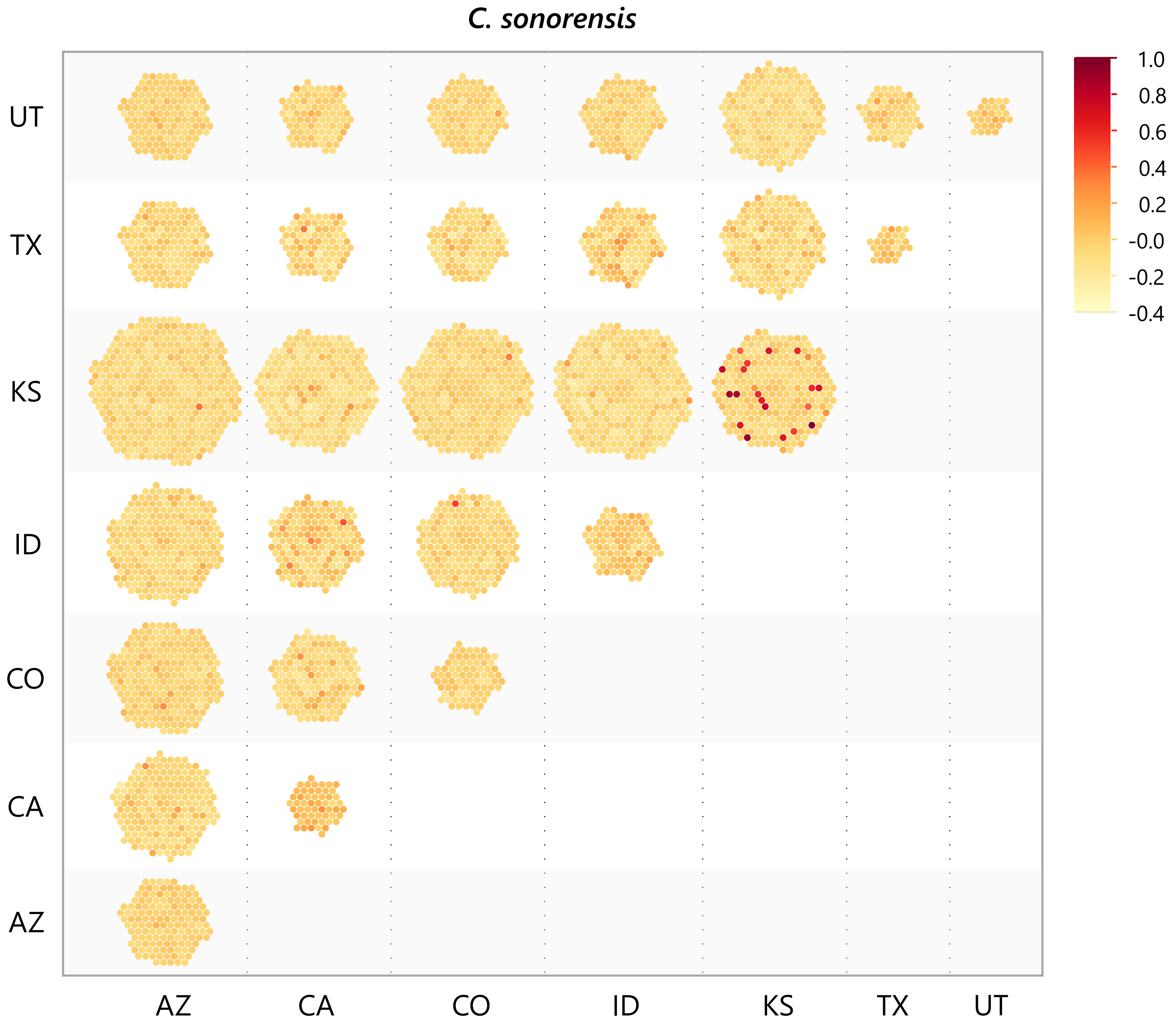

Supplemental Figure 6. Maximum likelihood estimation of relatedness between *C. sonorensis* individuals. Each circle represents the relatedness between two individuals compared across two populations (x and y-axis) with the diagonal representing the relatedness within a single population. Pairings with a higher relatedness coefficient score are considered to be more related and are shaded darker. This analysis was performed in R using the "relatedness" package (Laporte and Huard 2017).
